# Predicting irreversible electroporation-induced tissue damage by means of magnetic resonance electrical impedance tomography

**DOI:** 10.1038/s41598-017-10846-5

**Published:** 2017-09-04

**Authors:** Matej Kranjc, Simona Kranjc, Franci Bajd, Gregor Serša, Igor Serša, Damijan Miklavčič

**Affiliations:** 1 University of Ljubljana, Faculty of Electrical Engineering, Tržaška 25, 1000 Ljubljana, Slovenia; 2 Department of Experimental Oncology, Institute of Oncology Ljubljana, Zaloška 2, 1000 Ljubljana, Slovenia; 3 Jozef Stefan Institute, Jamova cesta 39, 1000 Ljubljana, Slovenia

## Abstract

Irreversible electroporation (IRE) is gaining importance in routine clinical practice for nonthermal ablation of solid tumors. For its success, it is extremely important that the coverage and exposure time of the treated tumor to the electric field is within the specified range. Measurement of electric field distribution during the electroporation treatment can be achieved using magnetic resonance electrical impedance tomography (MREIT). Here, we show improved MREIT-enabled electroporation monitoring of IRE-treated tumors by predicting IRE-ablated tumor areas during IRE of mouse tumors *in vivo*. The *in situ* prediction is enabled by coupling MREIT with a corresponding Peleg-Fermi mathematical model to obtain more informative monitoring of IRE tissue ablation by providing cell death probability in the IRE-treated tumors. This technique can potentially be used in electroporation-based clinical applications, such as IRE tissue ablation and electrochemotherapy, to improve and assure the desired treatment outcome.

## Introduction

Electroporation is a phenomenon that increases cell membrane permeability due to externally applied pulsed electric fields^1^. As most theoretical studies have predicted, the increase in cell membrane permeability is a consequence of structural changes that occur in the membrane in the form of hydrophilic pores^2, 3^. When the cell is exposed to an adequate electric field, transient structural changes can be attained^4, 5^. After a certain period of time, the membrane reseals and the cell survives. This is termed reversible electroporation as the cell preserves its viability. Medical applications of reversible electroporation, such as gene electrotransfer^6–10^ and electrochemotherapy^11–16^, exploit these structural changes in the membrane to increase the transmembrane transport of foreign DNA and chemotherapeutic drugs, respectively. In contrast, irreversible electroporation (IRE) induces cell death as the applied electric field is too strong for cells to recover, leading to excessive damage to cells and membranes. In recent years, nonthermal IRE for the ablation of solid tumors^17–20^ has emerged as a new medical technique. IRE has also been suggested for the treatment of atrial fibrillation as a nonthermal ablation method with minimal to no local heating effects^21–23^.

A cell can be exposed to an electric field by the application of electric pulses. Parameters of electric pulses, such as amplitude, duration and the number of pulses, play a major role in determining the outcome of all electroporation-based treatments, including IRE tissue ablation^2, 24, 25^. Therefore, the treatment efficacy of electroporation-based treatments, including IRE tissue ablation, can be enhanced by treatment planning, *i*.*e*. predicting electric field distribution through numerical modeling^26–30^. The outcome of IRE tissue ablation is usually evaluated by post-IRE medical imaging using either magnetic resonance (MR) imaging^31^, computed tomography^32^ or ultrasonography^33^. Even though the latter has been reported to be able to monitor IRE within minutes after intervention^34^, the main disadvantage of all the above-mentioned methods is the inability to monitor electroporation during pulse delivery. Since the efficiency of electroporation-based treatments is strongly correlated with electric field distribution^28, 35, 36^, a method capable of determining electric field distribution during the pulse delivery could potentially enable monitoring of electroporation-based treatments. This can be achieved by means of magnetic resonance electrical impedance tomography (MREIT), which enables measurement of electric field distribution during the application of electric pulses using MR imaging and mathematical algorithms^37, 38^. This method was recently demonstrated in mouse tumors *in vivo* to predict reversibly electroporated areas through measurement of the electric field distribution during the application of electric pulses^39^. In that study, however, the authors were only able to show a correlation between the electric field that led to reversible electroporation of tumor cells and the entrapment of contrast agent due to the electroporation. This was done by comparing tumor fractions, i.e., the size of the treated area divided by the size of the whole tumor, and not by direct comparison of either the size or shape of the treated areas. A direct comparison was not possible for technical reasons, such as the considerable time difference (24 h) between the treatment and the assessment of the contrast agent entrapment. Another limitation of that study was that the outcome of the treatment was predicted only based on the applied electric field distribution, i.e., by the amplitude of the electric field. In reality, it is not just the electric field that defines the outcome of electroporation treatments but also the duration of exposure to the field, i.e., the duration of a single electric pulse and the number of the applied pulses^40, 41^. For this reason, mathematical models have been developed to describe the effects of electroporation on the treated cell suspensions and tissues^42–45^. Our present study further improves MREIT-enabled electroporation monitoring of IRE-treated tumors by predicting IRE-ablated tumor areas during electroporation *in vivo*. This *in situ* prediction is enabled by coupling MREIT with a corresponding mathematical model to determine cell death probability in IRE-treated tumors.

## Materials and Methods

### Experimental design

IRE ablation of a murine tumor was achieved by application of electric pulses *via* two needle electrodes inserted into the tumor. First, a mouse with a tumor was placed in the MRI scanner and then scanned by the current density imaging (CDI) method during application of electric pulses in order to acquire a map of the magnetic field change induced by electric currents. The map was then processed according to Ampere’s law to obtain the corresponding current distribution map, which was then used to calculate the conductivity maps of the tumor using the MREIT algorithm. Finally, when current distribution and conductivity maps were determined, the electric field distribution in the tumor was calculated from the maps using Ohm’s law. Tumor tissue damage induced by IRE was then predicted by the Peleg-Fermi cell-death model using the measured electric field in the tumor and the number of applied electric pulses. After 72 hours, mice were sacrificed, and tumors were excised and fixed in zinc fixative and embedded in paraffin. Sections from each tumor were cut and then stained with hematoxylin and eosin (H&E) in order to determine necrotic areas, *i*.*e*. ablated areas due to IRE. The so-determined ablated areas of each tumor were then statistically compared with the corresponding predicted areas for the tissue damage given by the Peleg-Fermi cell-death model.

### Mice and Tumor Models

All animal experiments were conducted in accordance with the guidelines for animal experiments of the European Union directives, and permission was obtained from the Ministry of Agriculture and the Environment of the Republic of Slovenia (permission no. U34401-1/2015/7). Eleven-week-old female BALB/c mice were purchased from Charles River Laboratories Italia s.r.l. (Calco, Italy) and were maintained in specific-pathogen-free conditions with *ad libitum* access to food and water. Mice (20–21 g) were used in the experiments after eight days of adaptation. Subcutaneous tumors were induced by subcutaneous injection of 2 × 10^6^ mammary carcinoma TS/A cells in 50 µl of NaCl solution (0.9%, Braun Melsungen AG, Melsungen, Germany) in the left rear leg. The growth of tumors was followed by measurement of three orthogonal diameters (a × b × c) with a Vernier caliper. After six to seven days, when tumors grew to a volume of approximately 40 mm^3^, they were subjected to treatment.

Tumors were divided into two groups. The treated group consisted of nine tumors (m_1_–m_9_) that were subjected to the IRE treatment, while the control group consisted of two tumors (m_C1_, m_C2_) and only had the electrodes inserted but were not IRE treated.

### Irreversible electroporation treatment

**Figure 1:**
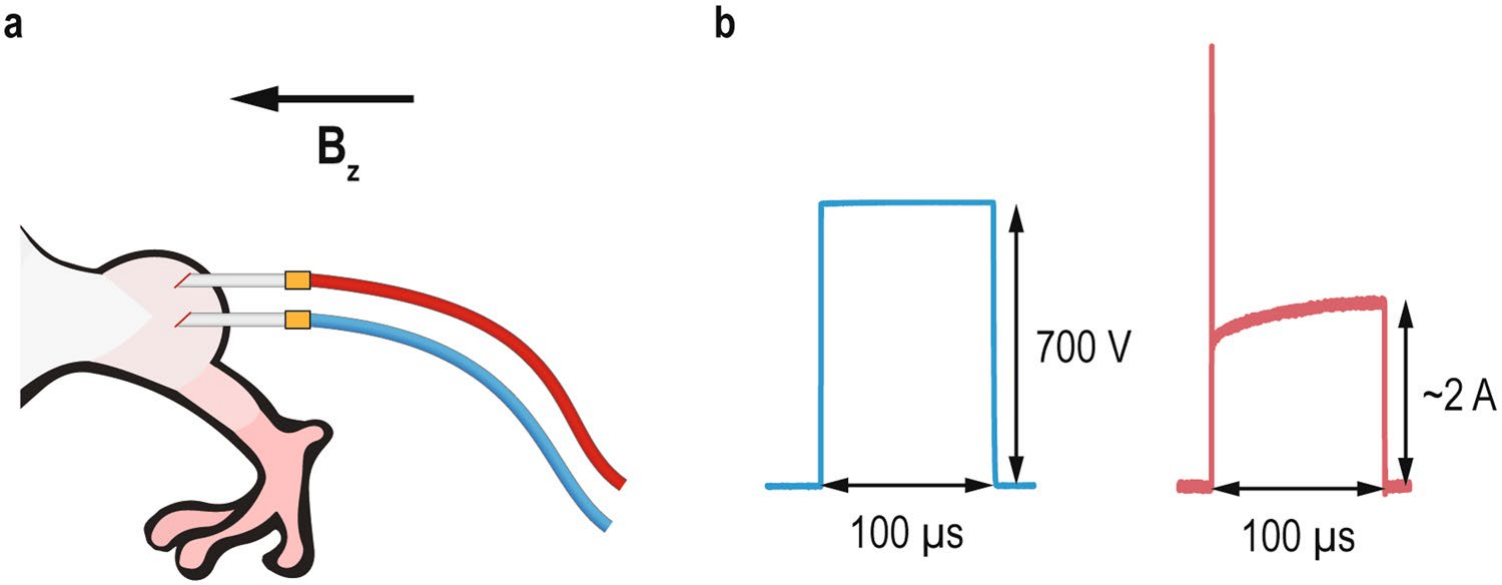
(**a**) Illustration of experimental *in vivo* setup for determining the electric field distribution during electroporation. Two needle electrodes were inserted into the tumor located on the mouse’s left rear leg. The electrodes were positioned in parallel with the static magnetic field of the MRI scanner (*B*_z_). (**b**) Measurements of the voltage (blue line) and the electric current (red line) of one of representative applied electric pulses.

Mice were initially anesthetized with an intraperitoneal injection of ketamine (1 mg/ml, Narketan®; Vetoquinol, Ittigen, Switzerland), xylazine (5 mg/ml, Chanazine; Chanelle Pharmaceuticals, Loughrea, Ireland) and acepromazine (0.4 mg/ml, Promace; Fort Dodge Animal Health, IA). IRE of mice tumors was performed by applying two sequences of four high voltage electric pulses (8 pulses altogether), each of 100 µs duration with a 700 V amplitude and with a pulse repetition frequency of 5 kHz (Fig. 1b). The electric pulses were delivered between two needle electrodes inserted into the tumor (Fig. 1a) by an electric pulse generator Cliniporator Vitae (IGEA s.r.l., Carpi, Italy). The needle electrodes were made of platinum-iridium (Pt/Ir: 99/1) and measured 25 mm in length and 0.5 mm in diameter. Center-to-center distance between the inserted electrodes varied between 1.2 mm and 3.3 mm, depending on the tumor size. The trigger input of the generator was connected to the MRI control unit and synchronized with the CDI pulse sequence. The outputs of the generator were also measured with an oscilloscope (WavePro 7300 A, LeCroy, Chestnut Ridge, NY, USA) and a current probe (AP015, LeCroy, Chestnut Ridge, NY, USA) to confirm the application of electric pulses.

### Magnetic Resonance Electrical Impedance Tomography

**Figure 2:**
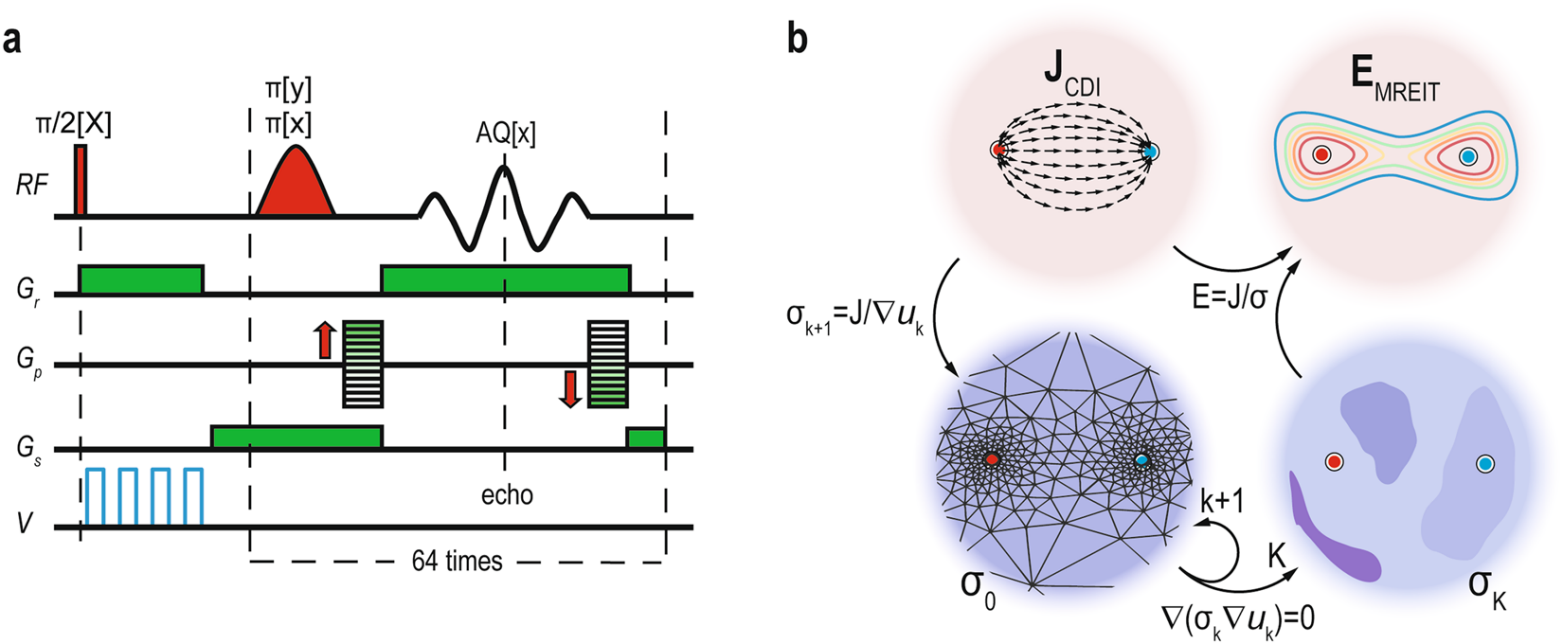
(**a**) Two-shot RARE CDI sequence that was used to acquire a map of current-induced magnetic field changes. The first part of the sequence is a current encoding part with four (100 μs long) high-voltage electroporation pulses (blue line) delivered immediately after the nonselective 90° radiofrequency (RF) excitation pulse. In the second part of the sequence, the image signal was acquired using the single-shot RARE signal acquisition scheme that includes standard execution of readout (*G*_r_), phase-encoding (*G*_p_) and slice-selection (*G*_s_) magnetic field gradients. (**b**) Electric field distribution E_MREIT_ in the tumor was calculated using Ohm’s law when an electric current density J_CDI_ and electrical conductivity σ_K_ were obtained. Electric current density in the tumor was determined using the current density imaging (CDI) method, while σ_K_ was obtained by means of J-substitution algorithm, i.e., an magnetic resonance electrical impedance tomography (MREIT) algorithm that was used for reconstruction of electrical conductivity inside a conductive body from current density data. The mathematical process of the algorithm is described in the Supplementary material. Electrodes are illustrated by the red circle and blue circle.

First, the CDI method was used to image current density in the tumor during the application of electrical pulses. The method relies on current-induced magnetic field changes in the sample that are detected via phase shift registration by magnetic resonance imaging^46, 47^. Next, a MREIT algorithm^48, 49^ (Fig. 2b), based on iterative solving of the Laplace equation, was employed to calculate a tumor conductivity map and electric field in the tumor by using CDI data along with known tumor geometry and potentials on the electrodes as inputs for the algorithm. In the study, two-shot rapid acquisition with relaxation enhancement (RARE) CDI sequence^50^ was performed on a 2.35 T MRI scanner (100 MHz proton nuclear MR frequency; Oxford Instruments, Abingdon, UK) equipped with microimaging accessories with maximum gradients of 250 mT/m (Bruker, Ettlingen, Germany). The following imaging parameters were used: field of view = 30 mm; imaging matrix = 64 × 64; inter-echo delay = 2.64 ms. In the sequence (Fig. 2a), the 700 µs-long block of electroporation pulses was positioned between the excitation RF pulse and the first refocusing RF pulse. The details of the current density imaging method and mathematical process of the MREIT algorithm for reconstruction of electric field distribution are described in the Supplementary material.

### Mathematical model for predicting tissue damage

**Figure 3:**
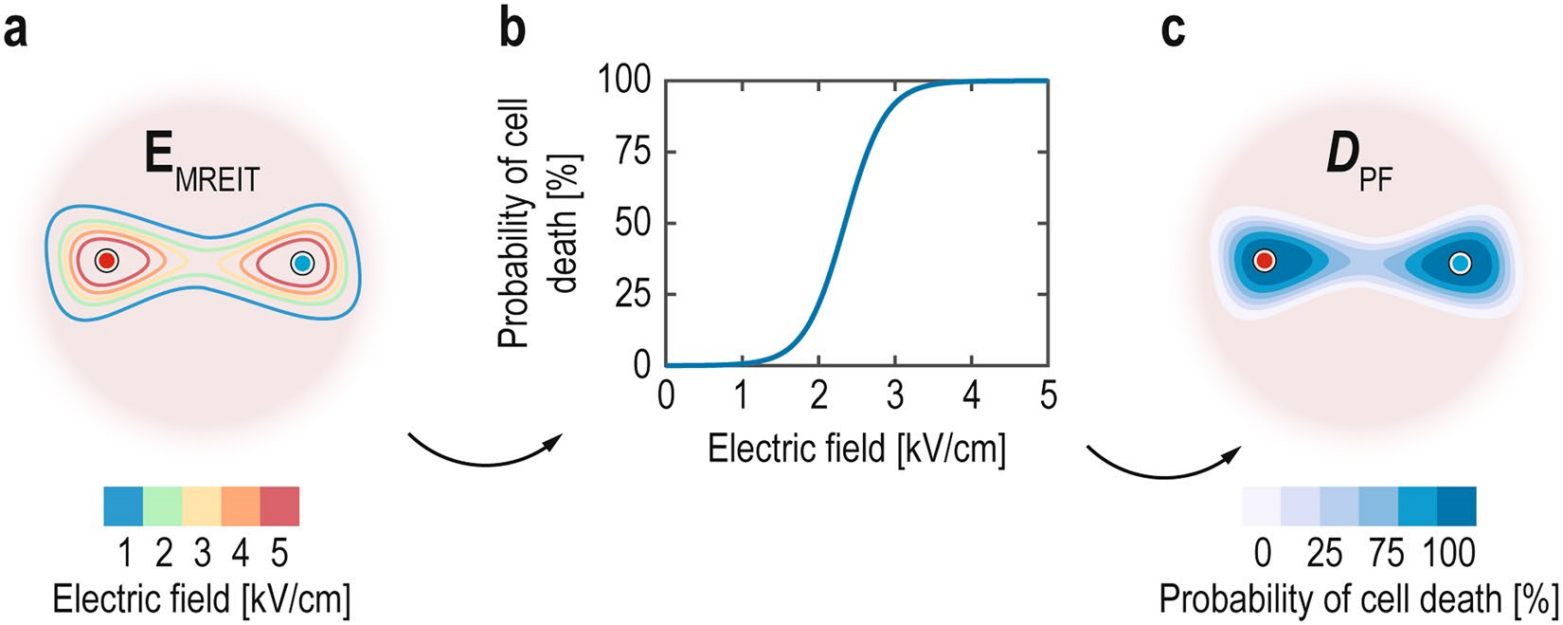
(**a**) Illustration of the electric field distribution in the tumor determined by the MREIT algorithm. Electrodes are illustrated as a red circle and blue circle. (**b**) Curve representing the probability of cell death with the increasing electric field for electric parameters of applied electric pulses (*N* = 8 and *t*_p_ = 100 µs) using the Peleg-Fermi model. (**c**) Illustration of the probability of cell death based on the Peleg-Fermi model and electric field distribution.

In this study, the Peleg-Fermi mathematical model^51^ was used for predicting tissue damage. The model includes dependency on the number of pulses as well as on the electric field and was successfully used in previous studies for describing cell death as a consequence of IRE^42–45^. In the Peleg-Fermi model, the probability of cell death (*D*_PF_) is given by:

$$\begin{array}{c}{D}_{{\rm{PF}}}(E,N,{t}_{{\rm{p}}})=1-S(E,N,{t}_{{\rm{p}}})\\ S(E,N,{t}_{{\rm{p}}})=\frac{1}{1+{e}^{\frac{E-{E}_{C}(N,{t}_{{\rm{p}}})}{k(N,{t}_{{\rm{p}}})}}}\end{array}$$

where *S* is cell survival, *E* is the applied electric field, *N* is the number of delivered electric pulses, *t*_p_ is the duration of single electric pulse, *E*_c_ is the critical electric field in which 50% of the cells are killed and *k* is the kinetic constant that defines the slope of the curve. Values *E*_c_ and *k* for different numbers and duration of electric pulses were empirically determined *in vitro* in a recent study on mathematical modeling of IRE-induced cell death^43^. For *N* = 8 and *t*_p_ = 100 µs, which were the electric parameters of electric pulses used in our study, the values of *E*_C_ and *k* were determined at 2.344 kV/cm and 0.2677 kV/cm, respectively^43^. The curve representing the probability of cell death with an increasing electric field for the determined *E*_c_ and *k* is shown in Fig. 3b. The Peleg-Fermi model was coupled with the MREIT algorithm for calculation of cell death probability (Fig. 3c) using electric field distribution determined in the tumor by means of MREIT (Fig. 3a).

### Histological Analysis

To obtain maximal tissue reaction (necrosis) after IRE, a preliminary experiment to estimate the necrotic area in tumors at 48 hours, 72 hours and 96 hours post-treatment was performed (results are available in Supplementary material). Due to the highest necrotic area observed at 72 hours after the IRE, the 72 hour timepoint was chosen for the sacrifice of mice. After the mice were sacrificed, tumors were excised and removed from underlying skin and fixed in zinc fixative (5 ml, BD Biosciences, San Diego, CA, USA). After 24 hours, the tumors were placed in 5 ml of 70% ethanol for an additional 24 h and finally embedded in paraffin. From the paraffin block in the middle of each tumor, 2 μm-thick sections were cut perpendicular to the insertion of the electrodes and stained with hematoxylin and eosin (H&E). The images of H&E-stained slides of the whole tumor were imaged with a DP72 CCD camera (Olympus, Hamburg, Germany) connected to a BX-51 microscope (Olympus Corporation, Tokyo, Japan). Necrotic areas in all H&E-stained slides were measured by one observer using CellSens Dimension software independently from the results obtained by MREIT.

### Statistical Analysis

All results were statistically analyzed using MATLAB 2016a and its Statistics and Machine Learning Toolbox (Mathworks, Natick, MA, USA). Linear regression was employed to assess the relationship between predicted IRE areas and ablated areas obtained by H&E staining. The slope and coefficient of determination (R^2^) were calculated for different probabilities of cell death. Bland-Altman analysis^52^ was applied to assess the agreement between predicted IRE areas and ablated areas within limits of agreement determined as the mean differences between predicted IRE areas and ablated areas ±1.96 standard deviation (95% confidence interval). A *p* value of less than 0.05 was considered to indicate statistical significance. The number of animals used for experiments was defined using Pearson correlation guidelines^53^.

## Results

**Figure 4:**
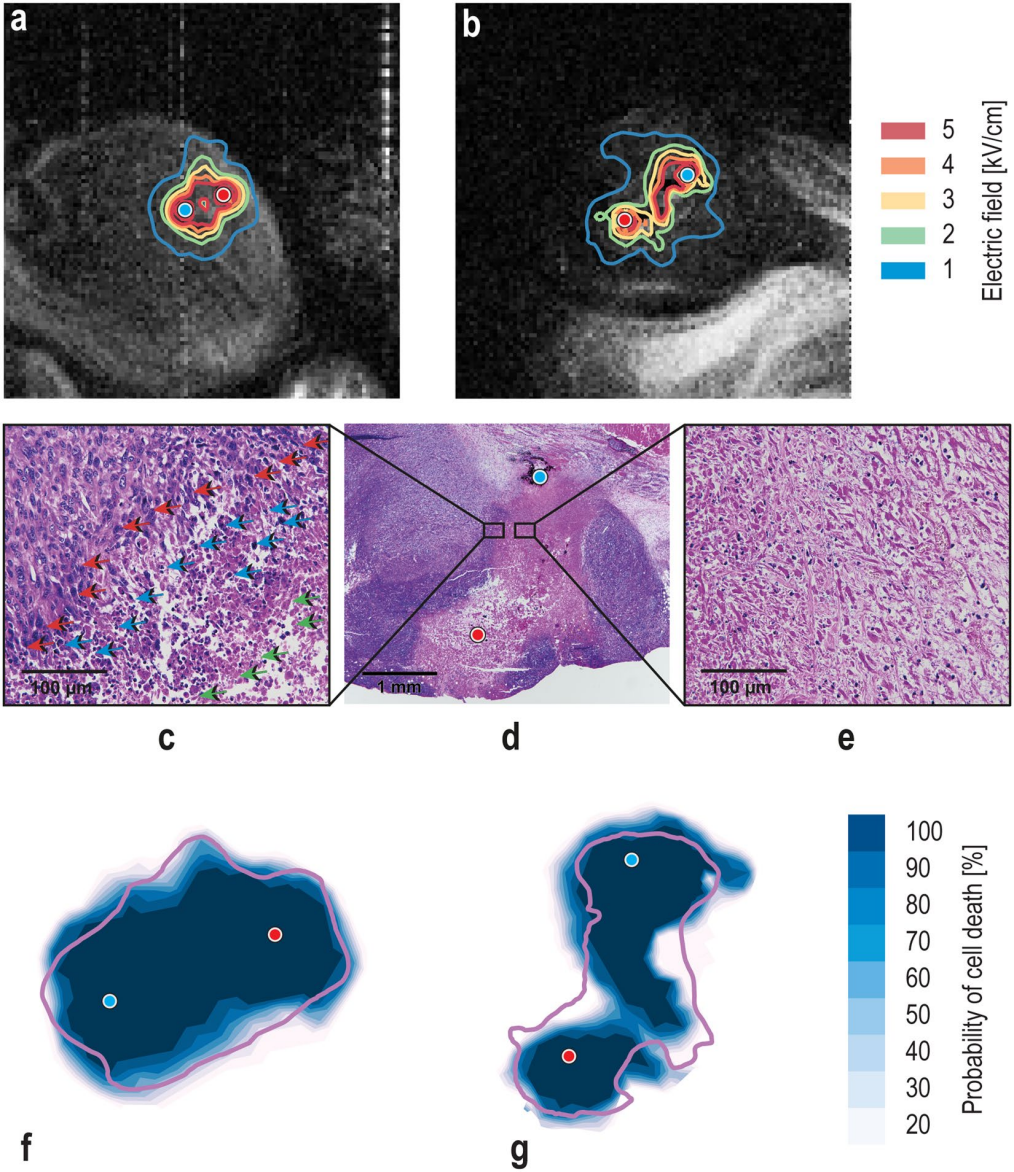
(**a**,**b**) Electric field distributions presented as contour plots in mouse tumor m_6_ (**a**) and m_7_ (**b**) obtained by means of MREIT. Contour plots are superimposed onto the corresponding T1-weighted images acquired before application of the electric pulses. Electrodes were inserted into the tumor perpendicular to the imaging slice and are illustrated as a red circle and blue circle. The distance between electrodes was 1.2 mm (**a**) and 2.2 (**b**) mm, and the applied voltage was 700 V. (**c**) 400× magnification of the margin area of the treated and untreated area of the *m*_7_ TS/A tumor tissue section after the IRE treatment. Green arrows depict necrotic cells in the treated area, blue arrows depict apoptotic cells at the edge of the treated area and red arrows depict normal tumor cells in the untreated area. (**d**) Representative image of the whole electroporated area in a TS/A tumor taken under 40× magnification. (**e**) 400× magnification of the necrotic area. (**e**,**f**) Comparison of the corresponding IRE areas predicted by the Peleg-Fermi model using determined electric field distribution obtained by MREIT and ablated zones obtained by H&E staining (purple solid line) in the tumors m_6_ (**e**) and m_7_ (**f**).

Electric field distributions were obtained in all tumors of the treated group (except in the control group since no electric pulses were delivered). Two examples of electric field distribution obtained by means of MREIT are shown in Fig. 4a and b, in which they were overlaid onto the corresponding T1-weighted image acquired just before the application of the electrical pulses. The highest electric field was established as expected near the electrodes, and it decreased with increasing distance from the electrodes.

The ablated zone was determined in H&E stained histological slides obtained from tumor samples 72 hours after IRE. Due to the small thickness of histological sections (2 μm), certain parts of the tumors were torn during preparation of the histological slides, especially in areas damaged by the electrodes. Images of tumor sections with nearly no torn parts are presented in Fig. 4c,d,e. The results demonstrate that cells in the tumors died of necrosis (Fig. 4e) in the treated area where the measured electric field was greater than 1.8 kV/cm (marked with green arrows, Fig. 4c). In the margin area, an extensive apoptosis was observed (marked with blue arrows, Fig. 4c). Necrotic areas in tumors of the control group were determined as the areas damaged by the insertion of the electrodes.

**Figure 5:**
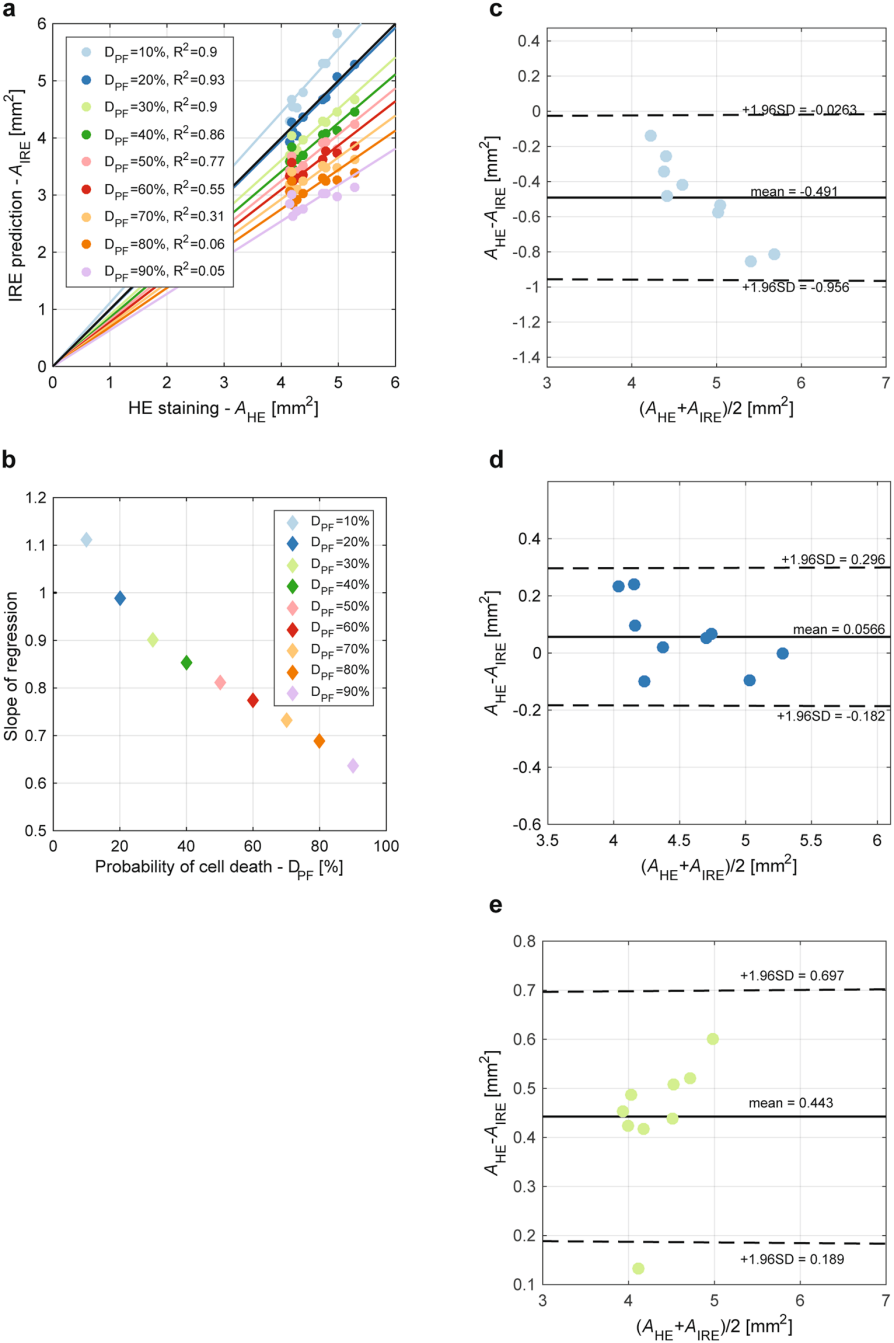
(**a**) Scatterplots of model-predicted areas in tumors (*A*_IRE_) obtained by means of the Peleg-Fermi model determined at different probabilities of cell death and the histologically determined ablated areas (*A*_HE_) obtained by H&E staining. Black solid line corresponds to the line of unity, whereas multiple color lines indicate regression lines of different slopes (**b**) that correspond to different cell death probabilities. (**c**,**d**,**e**) Bland-Altman plot for evaluating agreement between the model-predicted areas in tumors and the histologically determined ablated areas that correspond to cell death probabilities of 10%, 20%, and 30%, respectively. Dashed lines denote ±1.96 standard deviations (95% confidence interval).

Overlays of the predicted cell death probability of two tumors obtained by means of the Peleg-Fermi model with the corresponding histological slides are shown in Fig. 4f and g. Shapes of the model-predicted areas are similar to the shapse of the corresponding histologically determined ablated areas, whereas the extent of the model-predicted areas was comparable to the histologically determined ablated areas with lower values of cell death probability. To assess a relationship between the Peleg-Fermi model-predicted area (*A*_IRE_) and the histologically determined ablated area (*A*_HE_) for all tumors of the treated group, linear regression was employed to calculate regression slopes and the corresponding coefficients of determination (R^2^) for different probabilities of cell death (Fig. 5a and b). The regression line with the highest coefficient of determination (*R* ^2^ = 0.93, *p* < 0.001) and with a slope of 0.989 (Fig. 5a) corresponded to a 20% probability of cell death (Fig. 5b). Bland-Altman analysis in Fig. 5c–e, which was performed with 20% and with two adjacent cell death probabilities (10% and 30%), showed that the mean difference between *A*_IRE_ and *A*_HE_ was also the lowest with 20% cell death probability (0.0566 mm^2^, Fig. 5d). Here, it is expected from 95% confidence intervals that the difference between *A*_IRE_ and *A*_HE_ is in the range from −0.182 mm^2^ to 0.296 mm^2^. There was no significant correlation (*r* = −0.55, *p* = 0.12) between the difference on the y-axis and the mean on the x-axis, suggesting that the difference is approximately constant throughout the range of values obtained at a 20% probability of cell death.

## Discussion

In this study, the MREIT technique, which enables accurate determination of the electric field distribution in a treated tumor, was complemented with a Peleg-Fermi mathematical model for predicting tissue damage due to electroporation. Thus, an efficient method for prediction of ablated tissue areas in IRE-treated tumors was obtained. The method was tested in mouse tumors, and its results showed good agreement between tumor ablated areas predicted by the tissue damage model and those confirmed by histological analysis.

The prediction of ablated areas is strongly dependent on the IRE electric field threshold. As optimal electric pulse parameters depend on tissue susceptibility to electric pulses, different values of both irreversible as well as reversible electroporation thresholds have been reported in the literature. For IRE, a broad range of electric field strengths from 500 up to 2500 V/cm have been reported for different tissues^54–56^. In addition to a wide variety of tissues used in the studies, this large range of reported IRE thresholds is also a consequence of different applied electric pulse parameters. The electroporation outcome is not determined solely by the electric field strength but also by the exposure time to the field, i.e., the number and duration of electric pulses^40, 41^. In addition, electroporation cannot be regarded as a discrete effect where all cells exposed to an electric field above the threshold would be electroporated and those exposed to a field below the threshold would not be electroporated. In reality, there is a continuous effect with a broader transition interval of electric fields between the two states. The same characterization applies for the transition between reversibly and irreversibly electroporated tissue states. The so-called permeabilization and survival curves can thus be described using various mathematical models^43, 44^. In this study, the Peleg-Fermi model was used. The model was found to be efficient in the prediction of cell survival as a function of the electric field strength^43, 45, 51^. With implementation of the survival curves to the MREIT algorithm, a more informative monitoring of IRE tissue ablation was obtained. This monitoring also enables displays of cell death probability (Fig. 4f,g) instead of electric field distribution alone. Existing mathematical models of cell survival were developed on the basis of *in vitro* experiments, including the Peleg-Fermi model that was used in this study. Compared to cell suspensions, tissues *in vivo* have heterogeneous structure and consequently heterogeneous electrical conductivity, especially in tumors where conductivity can vary up to 20%^57^. Still, the heterogeneities, both original and those induced by the electroporation treatment, affect the determined electric field distribution^58^. Since *in vivo* survival curves are expected to have similar shapes as *in vitro* curves but lower electroporation thresholds^55^, the best agreement between histologically determined necrotic areas and those predicted by the cell death model was expected to be found at lower cell death probabilities. This was indeed confirmed, with the best obtained agreement at a 20% cell death probability. This agreement confirms that mathematical models for cell survival prediction *in vitro* present a good basis for studying cell death in tissues; however, in order to more precisely predict the cellular destruction *in vivo*, more accurate mathematical model parameters should be determined by further experiments on tissues *in vivo* using different amplitudes, durations, repetition frequencies and number of pulses. With these improvements, the models could potentially be used for real-time monitoring of IRE treatment, allowing possible treatment corrections in cases of insufficient tumor coverage with the electric field.

One of the limitations of this study is the 4 mm-thick MRI slices, which are much thicker than the 2 µm-thick histological sections. Thick MRI slices were needed to obtain sufficiently large image signals for reliable measurement of current distributions. With the use of a higher magnetic field magnet or longer electroporation exposure time, allowing signal averaging, the slice thickness could be reduced. Indeed, we have chosen to use 8 electric pulses to mirror previous studies on monitoring electric field distribution using MREIT, which is not a typical IRE sequence as it contains a sequence of 90 pulses or more, which by itself would allow thinner MRI slices. Thin histological sections also caused tearing of tumor sections, especially in areas damaged by the electrodes. Consequently, this prevented us from recovering the full shape of the treated areas on several of the histological slides and from performing statistical analysis of the similarity between the predicted and ablated areas.

In addition to electroporation-based treatments, there are also other applications that could benefit from the monitoring of electric field distribution. Another potent application is deep brain stimulation (DBS), an established technique to reduce neurological symptoms caused by numerous movement disorders, such as Parkinson’s disease and essential tremor^59^. Since the mechanisms of action are still uncertain, it is difficult to have complete control over the effects of DBS and to avoid undesired side effects. Therefore, mathematical models and simulations are commonly applied to DBS in order to obtain simulated stimulation fields that are visualized by the electric field distribution^60, 61^. It has been shown that the established electric field distribution during DBS is largely influenced by the heterogeneity of brain tissue, and the electric field distribution is therefore difficult to predict^62^. Indeed, MREIT was recently successfully applied for the monitoring of the current density distribution in the canine brain during DBS^63^ and it could be further expanded to obtain maps of established electric field distribution. Compared to IRE, the lower amplitude of established current density during DBS and different geometries of the electrodes would require the introduction of more advanced CDI methods, such as projected current density^63^.

In conclusion, the ability to predict irreversibly electroporated areas by means of MREIT and the Peleg-Fermi mathematical model are demonstrated. This method can potentially be used in electroporation-based clinical applications, such as IRE tissue ablation, where it can be applied for corrective interventions during the electroporation procedure and could therefore improve and assure the desired treatment outcome with complete tumor ablation. Furthermore, MREIT can provide valuable experimental results, such as IRE threshold determination and electric pulse parameters for treatment planning protocols, and for determining mathematical model parameters that can serve for prediction of damage in various tissues undergoing electroporation-based treatments.

## Electronic supplementary material

Supplementary material
