# Supplementary material for: Predicting irreversible electroporation-induced tissue damage by means of magnetic resonance electrical impedance tomography
Source: Sci Rep. 2017 Sep 4;7:10323. doi: 10.1038/s41598-017-10846-5 (PMC5583379; doi:10.1038/s41598-017-10846-5)
Supplement: Supplementary file 1 — Supplementary material [file 41598_2017_10846_MOESM1_ESM.pdf]

SUPPLEMENTARY MATERIAL

Predicting irreversible electroporation-induced tissue damage by means of magnetic resonance electrical impedance tomography

Matej Kranjc, Simona Kranjc, Franci Bajd, Gregor Serša, Igor Serša, Damijan Miklavčič

S.1. Methods

S1.1. Current Density Imaging

NMR/MRI is sensitive to any change in the magnetic field, as a change results in a shift of nuclear precession frequency. Additional magnetic fields to the main magnetic field of the NMR magnet can also be created by electric currents flowing through the sample. Not all components of the magnetic field are effective. Only the component in the direction along the main magnetic field is effective, as only this component adds to the main magnetic field and therefore changes the precession frequency. If currents flow in pulses, then the frequency shift is only temporary, i.e., only during the pulse. However, the effect of the pulse can be registered in the change of the nuclear precession phase, which can be expressed by

$$\varphi = \gamma B_{c\_z} t_p \ .$$

Here, γ is the gyromagnetic ratio of the observed nuclei (hydrogens), B_{c_z} is the magnetic field change along the main magnetic field direction induced by currents and t_p is the duration of the electric pulse. The equation can be reversed to express the magnetic field change $B_{c\_z} = \frac{\varphi}{\gamma t_p}$. In this way, the magnetic field change along the *z* direction (main magnetic field direction) can be measured. To calculate current distribution in the sample, all three components of the magnetic field change must be known. These can be measured by rotating the sample in perpendicular directions such that each time a different magnetic field change component is aligned with the main magnetic field. The entire magnetic field change vector can be measured as $\vec{B}_c = (B_{c\_x}, B_{c\_y}, B_{c\_z})$. Once the vector is known, current density in the sample during the electric pulse can be calculated by using Ampere's law:

$$\vec{j} = \frac{1}{\mu_0} \nabla \times \vec{B}_c \ .$$

Sample rotations in the magnet needed to obtain all magnetic field change components are generally difficult to perform or sometimes even impossible due to spatial confinements.

Another problem is also co-registration of the measured maps for their processing according to the Ampere's law equation. The rotations can sometimes be avoided in cases of special sample geometries. One such case is a situation when currents flow in a narrow plane. In this case, only the magnetic field change component that is perpendicular to the plane is dominant, and the other two (in plane) components are negligible. This is sufficient if the sample is oriented with the plane perpendicular to the main magnetic field such that only B_{c_z} is measured and current density components j_x and j_y are calculated simply as

$$(j_x, j_y) = \frac{1}{\mu_0}\left(\frac{\partial B_{c\_z}}{\partial y}, -\frac{\partial B_{c\_z}}{\partial x}\right).$$

The experimental conditions of current density imaging in mouse tumors presented in this study agree relatively well with the above example. In the experiment, the electrodes were aligned with the main magnetic field such that the current was flowing mainly in the perpendicular direction to the electrodes. The imaging plane was also oriented perpendicular to the main magnetic field and to the electrodes, and the plane was additionally positioned across the center of the tumor and central to the exposed part of the electrodes. Therefore, it is likely that currents were flowing mainly in the plane direction and that the corresponding magnetic field direction was mainly perpendicular to the plane.

S1.2. Two-shot RARE CDI sequence

In any electroporation experiment, it is important that the number of delivered pulses is adjusted to the treatment plan. Only the CDI sequences with fast signal acquisition are suitable for monitoring of the electric field established in the sample during delivery of electroporation pulses. With other CDI sequences that require several signal excitations and the associated delivery of extra sets of electric pulses, sample properties can change significantly during image signal acquisition. Ideally, the entire image signal is acquired after just one signal excitation using just one set of electroporation pulses.

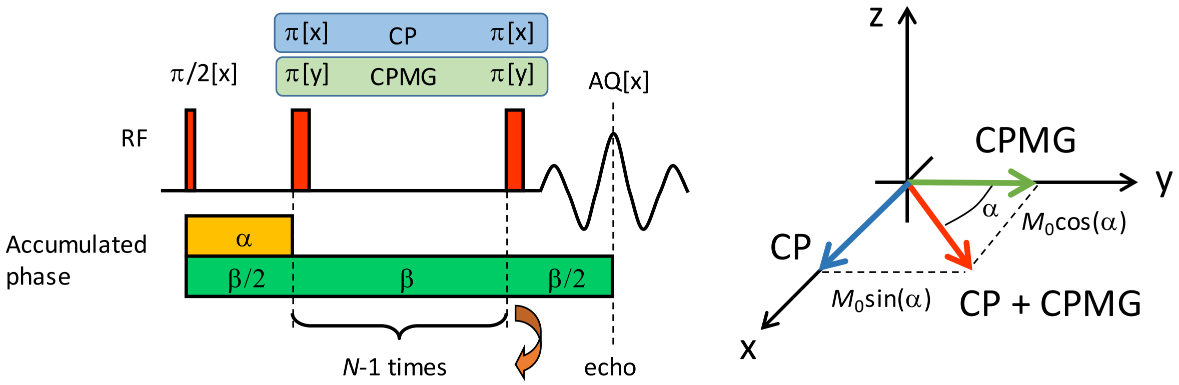

Supplementary Figure 1: Schematic presentation of the effect of electroporation pulses on the phase of an NMR signal in the two-shot RARE sequence. The accumulated phase has two contributions: one from electroporation pulses (yellow) and another from off-resonance spins (green). While the off-resonance contribution to the phase shift can be well compensated by using the CPMG scheme of RF pulses in the RARE sequence, the electroporation pulse contribution cannot be compensated by either the CPMG or CP scheme of refocusing pulses. These schemes of RF pulses lead to partial signals with magnetization refocusing along the *y*-axis (green, CPMG) or *x*-axis (blue, CP). However, co-addition of these two signals yields a complete signal (red, CP+CPMG), with a well-preserved current induced magnetization phase shift α.

In this study, a modification of the single-shot RARE (rapid acquisition with relaxation enhancement) sequence was used for electroporation monitoring. The sequence was modified by adding to it a block of electroporation pulses between the RF excitation pulse and the first RF refocusing pulse. When electroporation pulses are applied, the block results in an additional shift of the magnetization phase that makes the sequence unstable. With the sequence that uses standard phases of the refocusing pulses, which is [x] for the excitation pulse and [y] for all of the refocusing pulses, only the magnetization component oriented along the *y*-axis of the rotating frame contributes to the image signal. However, this is not sufficient for mapping of the magnetic field change and calculation of the corresponding current density image. Therefore, the single-shot RARE sequence needs another modification, which is a change of

the phase of all the refocusing pulses from [y] to [x]. With this modification, the magnetization component oriented along the *x*-axis of the rotating frame now contributes to the image signal, which is again not sufficient for current density image calculation. However, by co-adding these two incomplete image signals, one corresponding to the *x*- and another to the *y*-magnetization component, a complete image signal is obtained that enables mapping of the magnetic field change and calculation of the corresponding current density image. Due to two required signal excitations (sequence repetitions), the sequence is called the two-shot RARE CDI sequence.

S.1.3. Electric field reconstruction

Electric field distribution **E** in the tumor can be calculated using Ohm's law when an electric current density **J** and electrical conductivity σ are obtained:

$$\mathbf{E} = \frac{\mathbf{J}}{\sigma}$$

Electric current density **J** in the tumor was determined using current density imaging (CDI), while σ was obtained by means of the J-substitution algorithm, *i.e.* the magnetic resonance electrical impedance tomography (MREIT) algorithm used for reconstruction of electrical conductivity inside a conducting body by means of current density. First, a mathematical model of the tumor Ω_T was built based on segmented tumor boundaries obtained from T1-weighted images taken just before the application of electric pulses (Suppl. Fig. 2). Electrodes inserted in the tumor were modeled as two circular elements, Ω_{E+} and Ω_{E-} placed inside Ω_T.

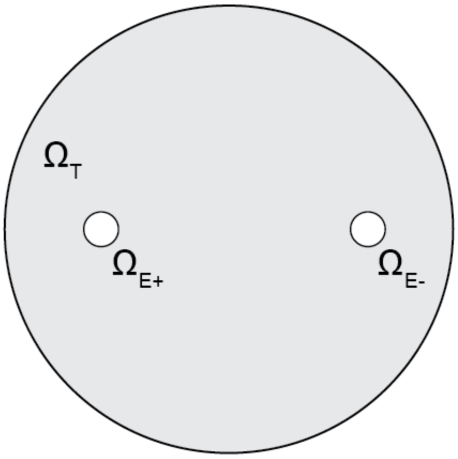

Supplementary Figure 2: A mathematical model of the tumor Ω_T and of positive Ω_{E+} and negative Ω_{E-} electrodes.

When electric pulses were applied, the following Laplace equation defined an established voltage u, satisfying the boundary value problem at any given σ of the Ω_T:

$$\nabla \cdot (\sigma \nabla u) = 0 \quad \text{in } \Omega_T$$

with the corresponding Neumann boundary condition on the boundary of Ω_T:

$$\sigma \frac{\partial u}{\partial n_T} = 0 \quad \text{on } \partial\Omega_T$$

and Dirichlet boundary condition on the boundary of Ω_{E+} and Ω_{E-}:

$$u = V \quad \text{on } \partial\Omega_{E+}$$

$$u = 0 \quad \text{on } \partial\Omega_{E-}$$

Since the Laplace equation consisted of two unknown variables, σ and u, the equation was solved by the iterative scheme:

$$\nabla \cdot (\sigma_k \nabla u_k) = 0 \quad \text{in } \Omega_T$$

$$\sigma_k \frac{\partial u_k}{\partial n_T} = 0 \quad \text{on } \partial\Omega_T$$

$$u_k = V \quad \text{on } \partial\Omega_{E+}$$

$$u_k = 0 \quad \text{on } \partial\Omega_{E-}$$

The iterative scheme started with a homogeneous sample conductivity (σ_k = const. where k = 0). The solution of the Laplace equation, *i.e.* u_k, was then used for calculation of the new conductivity σ_{k+1}:

$$\sigma_{k+1} = \frac{|\mathbf{J}_{\mathrm{CDI}}|}{|\nabla u_k|}$$

where $\mathbf{J}_{\mathrm{CDI}}$ is the current density distribution obtained by the CDI method. The iterative scheme continued until the relative differences between two successive σ_k and σ_{k+1} fell below 0.01. Finally, the electric field distribution was then determined using Ohm's law:

$$\mathbf{E} = \frac{\mathbf{J}_{\mathrm{CDI}}}{\sigma_k}.$$

MREIT was performed by using the finite element method with the numerical computational environment MATLAB 2016a (MathWorks, Natick, MA, USA) on a desktop personal computer.

S.1.4. Optimization of time for irreversible electroporation treatment effect visualization in histological sections

To observe the maximal necrotic tissue reaction to IRE treatment, a preliminary experiment to estimate the necrotic area in tumors at 48 hours, 72 hours and 96 hours post-treatment was performed. Mice and tumor models were the same as described in chapter 2.2. IRE of a mouse tumor was performed by applying two sequences of four high voltage electric pulses, each of 100 µs duration and with a pulse repetition frequency of 5 kHz. Two different amplitudes of pulses were applied, 700 V and 1000 V. Electric pulses were delivered between two needle electrodes by an electric pulse generator (Cliniporator Vitae (IGEA s.r.l., Carpi, Italy). Needle electrodes were made of platinum-iridium (Pt/Ir: 99/1) and measured 25 mm in length and 0.5 mm in diameter. Center-to-center distance between the inserted electrodes was between 1.2 mm and 3.3 mm, depending on the size of the tumor.

S.1.5. Determination of the necrotic area in tumors by histological analysis

To estimate the extent of necrotic areas in IRE-treated and in non-treated tumors, the animals were humanely sacrificed at 48 hours, 72 hours and 96 hours. Tumors were excised and removed from underlying skin, and fixed in zinc fixative for 24 hours (5 ml, BD Biosciences, San Diego, CA). Afterwards, the zinc fixative was exchanged with 5 ml of 70% ethanol, where tumors stayed for 24 hours before they were embedded in paraffin. Up to 20 consecutive 2 µm-thick sections from each tumor were cut perpendicular to the insertion of the electrodes from the paraffin block and stained with hematoxylin and eosin (H&E). The images of H&E-stained slides of the tumor were captured with a DP72 CCD camera (Olympus, Hamburg, Germany) connected to a BX-51 microscope (Olympus Corporation, Tokyo, Japan). Necrotic areas in tumor sections were measured by a single observer using CellSens Dimension software (Olympus).

S.2. RESULTS

S.2.1. Necrotic area in tumor

Necrotic areas were determined in all tumor sections of untreated (control) and IRE-treated tumors. (Supplementary Table 1). In untreated tumors, the determined necrotic area was comparable at all time points of observation. The treatment of tumors with IRE using 1000 V caused up to 97.5% of the area between the electrodes to become necrotic, and the necrosis also extended into tissue areas that were not exposed to the electric field. Treatment of tumors with IRE at the lower voltage, 700 V, induced a lower percentage of necrosis (up to 81.9%) between the electrodes and did not affect the tissue outside of the area exposed to the electric field. Therefore, in order to focus on the tissue ablation only between the electrodes, an electric pulses protocol with the lower voltage (700 V) was used for the study. In addition, to estimate the correlation between ablated zones only between the electrodes (or tissues exposed to the electric field) obtained by H&E staining with the imaging using MREIT, the 72-hour post-treatment interval was chosen for further investigation (Supplementary Table 1, Supplementary Fig. 3).

Supplementary Table 1: Necrosis in TS/A tumors after IRE at 700 V and 1000 V determined at different time points

**Group**	**N**	**48 h** (AM ± SEM)	**72 h** (AM ± SEM)	**96 h** (AM ± SEM)
**Control**	3	6.5 ± 2.1	5.8 ± 0.8	4.2 ± 1.4
**700 V**	3	45.0±12.9	81.9 ± 2.8	39.6±11.2
**1000 V**	3	64.2.±11.9	97.5±2.5	80.6±9.2

Abbreviations: AM- arithmetic mean; SEM- standard error of arithmetic mean

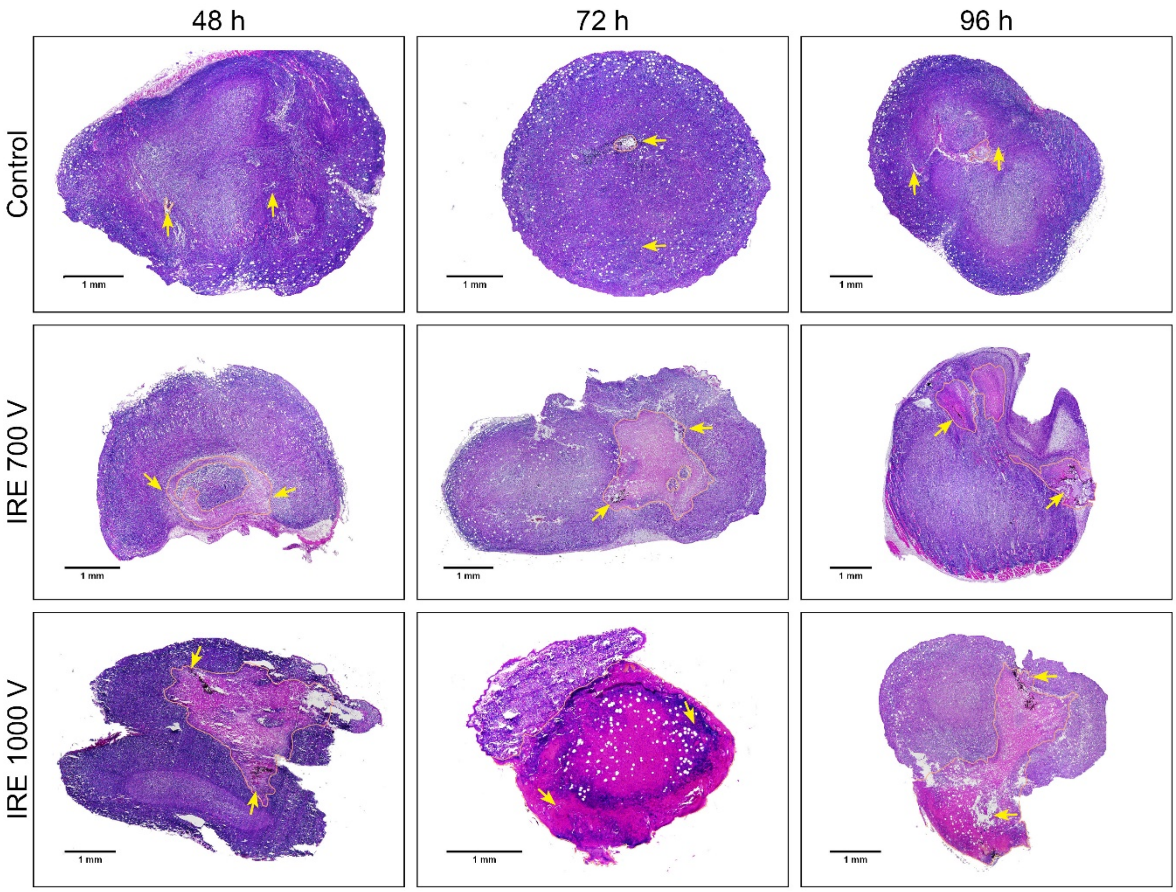

Supplementary Figure 3: Necrotic area in TS/A tumors after treatment with IRE. Yellow arrows depict the location of electrodes; the orange solid line encloses the necrotic area.
